# Balancing Pain Relief and Safety: Gastrointestinal and Cardiovascular Risk Assessment in Nonsteroidal Anti-Inflammatory Drug Users and the Role of Gastroprotective Co-Therapy

**DOI:** 10.3390/ph19010067

**Published:** 2025-12-29

**Authors:** Javedh Shareef, Sathvik Belagodu Sridhar, Zainab Mohamed Saeed, Amal Mohamed Rashed Alsereidi

**Affiliations:** Ras Al Khaimah College of Pharmacy, Ras Al Khaimah Medical and Health Sciences University, Ras Al Khaimah 11172, United Arab Emirates; phzainab3939@hotmail.com (Z.M.S.); amal.25928001@rakmhsu.ac.ae (A.M.R.A.)

**Keywords:** chronic pain, non-steroidal anti-inflammatory drugs, risk factors, prescriptions, pharmacy

## Abstract

**Background/Objectives**: Nonsteroidal anti-inflammatory drugs (NSAIDs) are widely used for pain management but pose gastrointestinal (GI) and cardiovascular (CV) risks, particularly during long-term use. This study evaluated NSAID-prescribing patterns and the appropriateness of gastroprotective co-therapy among patients with varying GI and CV risk profiles. **Methods**: An observational, cross-sectional study was conducted in the outpatient pharmacy department over six months (March 2023 to August 2023) at a public secondary care facility. Data pertaining to patient demographics, NSAIDs prescription, and GI/CV risks were collected and reviewed from electronic health records. Descriptive statistics, chi-square tests, and logistic regression were performed. **Results**: A total of 1005 prescriptions containing 2051 NSAIDs were analyzed. Selective COX-2 inhibitors and non-selective NSAIDs were the most frequently prescribed. Only 42.1% of patients received proton-pump inhibitors despite guideline recommendations. Non-selective NSAIDs were significantly associated with CV history and GI risk (*p* < 0.0001). Logistic regression showed age, gender, CV history, and GI risk significantly influenced NSAID selection. Notably, non-selective NSAIDs continued to be prescribed among moderate- and high-GI-risk patients. **Conclusions**: Suboptimal adherence to guideline-recommended gastroprotective strategies was evident, particularly among high-risk patients. Comprehensive GI and CV risk assessment and the rational use of gastroprotective co-therapy are essential. Integrating evidence-based digital tools may enhance safer NSAID prescribing in routine practice.

## 1. Introduction

Globally, Nonsteroidal Anti-inflammatory Drugs (NSAIDs) remain the preferred frontline therapy for managing pain and inflammation disorders [1]. Documented extensively for their analgesic and anti-inflammatory properties, they are widely used to control symptoms, ranging from common ailments to more complex disorders, including musculoskeletal disorders, acute and chronic injuries, and postoperative recovery [2]. They remain an integral component in modern healthcare management due to their well-established therapeutic efficacy, cost-effectiveness, and ease of access [3]. The growing concern over adverse drug reactions and hospital admissions associated with NSAID use raises important questions about their safety profile, despite their proven clinical utility [4]. The chronic and prolonged use of NSAIDs has been associated with notable negative effects, especially in subjects with a history of gastrointestinal (GI) disorders, such as inflammatory bowel disease (IBD), gastric ulcers, or gastroesophageal reflux disease (GERD) [5,6]. Furthermore, extended use in individuals with pre-existing cardiovascular (CV) or kidney problems may exacerbate their underlying cardiac and renal risks [3,7].

GI toxicity continuous to remain one of the most frequent reported adverse effects associated with NSAID use. The inhibition of cyclooxygenase (COX) enzymes, particularly COX-1, reduces the mucus and bicarbonate secretion, thereby weakening the stomach lining’s defenses, mucosal blood flow, and epithelial repair and predisposing the stomach for more susceptible damage from gastric acid [8]. The literature reports that nearly 30% of long-term NSAID users’ complaints about symptoms were related to dyspepsia, while approximately 1–2% experienced chronic complications, such as bleeding and perforation, every year [2,5,6]. In some populations, the likelihood of GI problems is quite high [9]. Severe GI events are more prevalent among elderly patients, individuals with a record of GI ulcers, and those who are using NSAIDs alongside antiplatelet medications or corticosteroids. Furthermore, the likelihood of bleeding complications is significantly heightened when NSAIDs are combined with alcohol or anticoagulants [10].

In addition to GI issues, the CV safety of NSAIDs has gained significant attention in recent years. The use of nonselective NSAIDs, such as ibuprofen and diclofenac, and certain selective NSAIDs, especially in patients with pre-existing CV diseases, has been linked to an increased risk of CV diseases and stroke [11,12]. This necessitates frequent and long-term monitoring in these high-risk groups as they are more prone to use NSAIDs to treat chronic illnesses, like osteoarthritis or musculoskeletal pain [9]. Therefore, it is very important that the prescriber Performa thorough GI and CV risk assessment and an overall benefit–risk profile of each NSAID and personalize the treatment according to each patient’s characteristics [4,6].

Various international guidelines published across the globe also supported the above-mentioned remarks and underscore the need for vigilant prescribing, mainly in individuals with these pre-existing risks [9,10,13]. The co-prescription of gastroprotective medications, such as proton pump inhibitors (PPIs) or H2 receptor blockers, or misoprostol, is a crucial part of risk management because they may mitigate the adverse GI effects associated with NSAIDs in patients identified as high-risk for bleeding or gastric ulcers [14]. Nevertheless, despite explicit recommendations supporting their usage, evidence suggests that gastroprotective medicines are underutilized in several patient populations, making this practice frequently inconsistent [10].

Therefore, assessment of prescribing practice becomes vital in bridging the gaps between therapeutic guidelines and real prescribing practice. Analyzing the integration of GI and CV risk assessment by the prescribers into clinical decision-making sheds light on the quality of care and identifies any areas for interventions. Additionally, incorporating evidence-based digital platforms, such as computerized drug decision support systems (CDDS), represents a transformative approach to systematically analyzing the risk assessment, identifying drug-related problems, and choosing the best alternatives or recommending protective strategies. Evidence indicates that using these technologies significantly improves prescribing safety and clinical outcomes in complex medication regimens [15].

In the healthcare settings of the UAE, the systematic evaluation of prescribing trends and gastroprotective strategies becomes essential when both prescription and non-prescription NSAIDs are easily available. Variations in patient comorbidities, prescribing awareness, and access to institutional support systems may influence the quality of NSAID use. Therefore, this study aimed to evaluate NSAID-prescribing patterns, assess the appropriateness of gastroprotective co-therapy in patients with varying gastrointestinal and cardiovascular risk levels, and identify the factors influencing prescribers’ NSAID selection in routine clinical practice.

## 2. Results

A total of 1005 prescriptions were analyzed to assess NSAID-prescribing patterns alongside GI and CV risk factors. The demographic characteristics of the cohort are summarized in Table 1. Overall, the sample included slightly more females than males, with a mean age of 46.9 years. Nearly half of the patients were between 26 and 50 years of age, and the majority were UAE nationals. More than half of the cohort had at least one comorbidity, with cardiovascular conditions and diabetes being the most common. Polypharmacy was prevalent, with most patients receiving between one and five medications (Table 1).

### 2.1. Underlying Clinical Indications for the Use of NSAIDs in Study Patients

After analyzing the different diagnoses for prescribing various NSAIDs, musculoskeletal and mechanical pain constituted the most common indication (61.98%), followed by dental and ENT-related pain (14.46%) and inflammatory joint disorders (11.81%). The clinical indications for NSAID use are summarized in Table 2.

### 2.2. Prescribing Pattern of NSAIDs

Among 1005 patients, a total of 2051 NSAID prescriptions were recorded, corresponding to a mean of 2.04 ± 0.84 NSAIDs per prescription. Both oral and topical NSAID formulations were prescribed in more than half of the patients (57.01%), while oral-only NSAIDs were used in 40.89% and topical-only NSAIDs in 2.08% of cases; fixed-dose combinations of paracetamol with orphenadrine citrate accounted for 12.48% of prescriptions. Selective COX-2 inhibitors constituted nearly 23.74% of all NSAID prescriptions. The distribution of NSAID prescriptions by therapeutic class, dose, and frequency of administration is shown in Table 3.

### 2.3. Co-Prescribing Gastroprotective Agents with NSAIDs

In the study sample, only 42.1% of patients received prescriptions for PPIs, such as Omeprazole (50.4%), Dexlansoprazole (26.6%), and Esomeprazole (17.4%), in conjunction with NSAIDs. Conversely, 57.9% of patients did not receive any GI protective agent. Among them, 25.3% of the patients received COX-2 inhibitor prescriptions. Consequently, 32.7% of patients received COX-1 inhibitors without any GI protective agent.

### 2.4. Impact of GI Risk Factors with NSAIDs Prescribed

The analysis of GI risk associated with prescribed NSAIDs indicates that 490 patients, representing 48.75%, were on long-term NSAID therapy, which may elevate the GI risk for these individuals (*p* = 0.018). A total of 141 patients, representing 14.02%, were identified as aged 65 years and above, and this age group showed a statistically significant association with higher GI risk categories (*p* < 0.0001). Among the comorbidities, 224 (22.28%) patients with CV diseases exhibit an increased GI risk associated with prescribed NSAIDs (*p* = 0.005). The significance reflects the observed variation in patient characteristics across GI risk categories, consistent with the results summarized in Table 4.

### 2.5. Prescribed NSAIDs and Risk of CV Outcomes

As advised in the guidelines released by the international NSAID consensus committee, previous CV incidents and persistently elevated blood pressure are the risk factors for CV outcomes that limit the use of NSAIDs.

**Figure 1:**
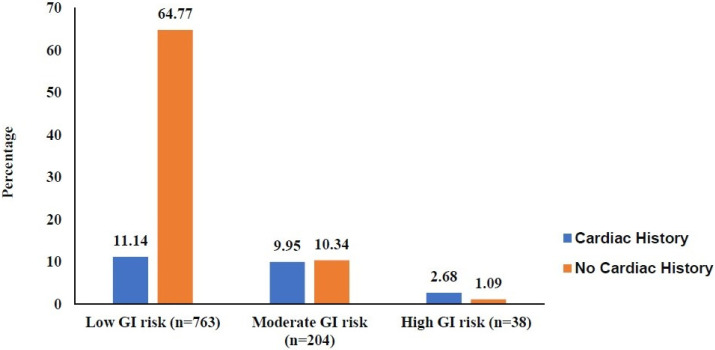
GI and CV risk in the study populations.

Figure 1 illustrates the distribution of patients across the three GI risk categories—minimal, moderate, and high—stratified by cardiovascular history. Among patients with minimal GI risk, the majority had no CV history (80.21%), with only a small proportion having a CV history (19.78%). In contrast, patients with moderate GI risk showed a more balanced distribution between those without CV history (53.9%) and those with CV history (46.08%). In the high GI-risk group, a considerable proportion had a CV history (55.26%), compared with those without a CV history (44.73%). Overall, the figure demonstrates that the prevalence of combined GI and CV risks increases progressively across the risk categories, emphasizing the clinical importance of assessing both factors before prescribing NSAIDs.

### 2.6. Appropriateness of Drug Use Based on the GI and CV Risk Factors Among the Study Populations

When examining medication therapy based on the patient’s level of GI risk, a statistically significant link was found between the prescription of NSAIDs or a combination of NSAIDs and a GI protective agent, and the degree of the patient’s GI risk (*p* < 0.0001). Patients categorized as having a moderate-to-high GI risk had a higher probability of being prescribed non-selective NSAIDs alone or with GI protective agents than patients with low GI risk (7.35% and 8.82% versus 6.42%, respectively, *p* < 0.0001). Similarly, patients with moderate-to-high GI risk were more likely to be prescribed preferential COX-2 inhibitors in the absence of a GI protective agent (*p* < 0.05) (Table 5).

Regarding the patient’s GI risk profile and CV history, the evaluation of NSAID prescriptions alongside the GI and CV risks of patients revealed a notable difference between the use of Nonselective NSAIDs (NsNSAIDs) with or without gastroprotective agents. Similarly, regardless of their CV history, those with moderate to high GI risk were prescribed more favorable preferential COX-2 inhibitors and selective COX-2 inhibitors, regardless of GI protective agents; nevertheless, the results fail to achieve statistical significance (Table 6).

A logistic regression analysis was conducted to determine the relationship between several factors (such as age, gender, comorbidities, GI risk levels, etc.) and the prescription of various types of NSAIDs, including those combined with PPIs. Older age and a higher GI risk category were significantly associated with increased odds of receiving selective COX-2 inhibitors (*p* < 0.05). In contrast, male gender and the absence of CV history were predictors of prescribing non-selective NSAIDs. Preferential COX inhibitors were not significantly associated with any demographic variable (Table 7).

### 2.7. Paracetamol

Individuals diagnosed with neurological conditions exhibited a decreased probability of being prescribed paracetamol (OR: 0.388, 95% CI: 0.149—1.000, *p* < 0.05) in contrast to those not affected with such conditions.

### 2.8. Nonselective NSAIDs

Nonselective NSAIDs were more frequently prescribed for the age group between 50 and 64 years (OR: 3.422, 95% CI: 1.261–9.289, *p* < 0.05), indicating they had a threefold higher chance of receiving these medications compared to patients less than 50 years old. Conversely, these medications were at decreased odds of being prescribed to patients with moderate (OR: 0.078, 95% CI: 0.027–0.219, *p* < 0.001) and high risk (OR: 0.122, 95% CI: 0.054–0.274, *p* < 0.001), reflecting a careful strategy to lower the risk of GI problems in these groups of patients.

### 2.9. Nonselective NSAIDs with Gastroprotective Agents

NSAIDs combined with gastroprotective agents (PPI) were more frequently prescribed to patients with CV conditions (OR: 2.363, 95% CI: 1.301–4.289, *p* < 0.05), in contrast to those not affected with such conditions. This indicates that prescribers exercised greater caution when prescribing these medications due to the potential for heightened CV risks linked to nonselective NSAIDs.

Patients presenting with moderate (OR: 0.035, 95% CI: 0.009–0.130, *p* < 0.001) and high GI risks (OR: 0.061, 95% CI: 0.023–0.165, *p* < 0.001) were less likely to receive prescriptions for non-selective NSAIDs with PPI, as clinicians tended to favor safer alternatives to mitigate the risk of GI complications.

### 2.10. Preferential COX-2 Inhibitors

Males were more likely to be prescribed preferential COX-2 inhibitors (OR: 1.431, 95% CI: 1.097–1.868, *p* < 0.05), in comparison to female patients.

### 2.11. Preferential COX-2 Inhibitors with a GI Protective Agent

The analysis indicated that patients categorized with moderate risk (OR: 0.271, 95% CI: 0.087–0.844, *p* < 0.05) were less likely to receive prescriptions for preferential COX-2 Inhibitors in comparison to those with low GI risk.

### 2.12. Selective COX-2 Inhibitors

COX-2 inhibitors were less frequently prescribed to males (OR: 0.718, 95% CI: 0.558–0.923, *p* < 0.05) in comparison to females. However, they were more likely to be prescribed in moderate GI risk groups OR: 2.847, 95% CI: 1.077–7.524, *p* < 0.05) in comparison to patients with low GI risk. This indicated that the prescribers’ preferred these options due to their superior safety profile regarding GI complica-tions compared to other NSAIDs.

### 2.13. Selective COX-2 Inhibitors with a Gastroprotective Agent

Male patients exhibited a lower likelihood of being prescribed selective COX-2 inhibitors alongside a PPI (OR: 0.724, 95% CI: 0.540–0.972, *p* < 0.05), compared to female patients. This finding suggests that prescribers may exercise greater caution when prescribing these combinations to male patients, possibly due to clinical factors such as comorbidities and social history, in order to minimize the risk of NSAID-related gastrointestinal or cardiovascular complications.

## 3. Discussion

The effective management of pain mainly depends on the rational and appropriate use of NSAIDs in healthcare settings. The current research demonstrates a higher prescription pattern for non-selective COX inhibitor NSAIDs, which aligned with findings from other research conducted in comparable hospital environments [8,11,13,16]. The pattern of NSAID prescription differed across the studies, with most of them confined to the orthopedic department [17,18,19,20]. In contrast, our study collected prescriptions from a hospital pharmacy department receiving orders from various clinical specialties. However, most of the prescriptions were still from orthopedic departments compared with other specialties. This could be attributed to the higher number of patients who visited orthopedic departments with complaints of back ache and/or joint pain/arthritis, and NSAIDs are commonly prescribed to manage such clinical conditions. Ibuprofen was the most frequently prescribed Non-selective COX inhibitor NSAID. The high-frequency non-selective or traditional NSAID prescription patterns noted in this study are consistent with earlier studies in similar hospital settings [21,22,23,24].

Interestingly, celecoxib emerged as the single and most frequently prescribed selective COX-2 inhibitor among our study population. The desirable safety profile, coupled with a superior efficacy in managing pain than conventional NSAIDs (as reported in earlier studies), may explain their frequent prescription [25]. Additionally, it offers the benefits of a prolonged effect and a reduced incidence of GI side effects commonly associated with traditional NSAIDs, positioning coxibs as a more favorable therapeutic choice. The PRECISION trial indicated that selective COX-2 inhibitors are associated with lower risks of GI issues and CV events compared to non-selective NSAIDs, such as ibuprofen and naproxen [26].

The assessment of co-prescribing PPIs alongside NSAIDs indicated that fewer than half of the study populations received gastroprotective agents, while the majority did not receive co-therapy. This finding points to suboptimal adherence to recommended gastroprotective practice. Similar trends were observed in the previous studies, indicating that gastroprotective agents were prescribed in suboptimal proportions relative to the total NSAID prescriptions [27,28]. However, in contrast to our findings, another study reported a higher prevalence (72%) of gastro-protective agent co-prescription along with the NSAIDs [29]. The selection of a gastroprotective agent among the PPIs is likely influenced by specific patient characteristics, including cost considerations, insurance coverage, and the preferences of the prescribing physician. International studies have similarly highlighted concerns regarding the quality of NSAID prescribing, particularly related to adherence to GI and CV safety guidelines. Studies from Europe, the United States, and Asia have reported the inconsistent use of gastroprotective agents among high-risk patients and frequent prescribing of non-selective NSAIDs despite clear cardiovascular risks. These global findings parallel our results and underscore the widespread challenge of ensuring evidence-based NSAID prescribing in routine clinical practice.

The analysis of the prescriptions without gastroprotective agents showed that the majority involved COX-2 inhibitors, while a smaller proportion included other NSAIDs prescribed without PPIs. These patients are likely to remain at a higher risk of experiencing GI complications. Therefore, judicious NSAID use and careful consideration of concomitant medications are essential to prevent or minimize potential adverse effects.

To promote evidence-based practice and support physicians in ensuring appropriate NSAID prescribing across diverse patient populations, international prescribing guidelines have been established and integrated into routine clinical practice [30]. However, the extent to which these recommendations have been complied with or integrated effectively into routine clinical practice remains uncertain. The primary purpose of these guidelines is to help prescribers optimize and improve the prescribing accuracy, enhancing the quality of care and mitigating NSAID-related adverse outcomes, particularly among high-risk patients [31]. It is well known that the elderly are more susceptible to NSAID-related adverse effects due to their frequent use and age-related comorbidities [27]. Patients with both significant GI and CV risk factors, as well as those with high GI risk alone, were observed to have the most pronounced gaps in adherence to therapeutic recommendations [32]. Concurrent with this evidence, our study findings make evident that non-selective NSAIDs or preferential COX-2 inhibitors were predominantly prescribed among patients at moderate to high GI risk. These drugs should generally be avoided or used with extreme caution in such groups, as this could escalate the risk of GI complications, including ulcers, bleeding, and perforation. Notably, the use of non-selective NSAIDs co-prescribed with PPIs among patients with high GI risk reflects a comparatively safer and more guideline-concordant approach than prescribing NSAIDs alone. This practice aligns with the recommendations of the International NSAID Consensus Group and indicates an increasing level of prescriber vigilance regarding gastroprotection in high-risk populations, as also reported in previous studies [31,33].

Furthermore, prescribing restrictions should be applied for non-selective NSAIDs in high-risk individuals, particularly those with GI risk factors and CV history. The statistically significant *p*-value (<0.0001) indicates that non-selective NSAIDs were disproportionately prescribed to patients with higher GI and CV risks, despite guideline recommendations. Such prescribing practices deviate from standard guidelines and should be avoided or replaced with safer alternatives, such as selective COX-2 inhibitors with appropriate gastroprotection. In contrast, prescribing preferential COX-2 inhibitors in patients presenting with low-to-moderate GI risk and a history of CV issues does not elicit immediate concerns, as the utilization of NSAIDs in low-risk patients is typically deemed rational. Although the finding was not statistically significant, the use of preferential COX-2 inhibitors, either alone or in conjunction with a gastroprotective agent, remains clinically relevant in patients at high risk for GI complications, warranting careful consideration due to the potential for serious complications. Selective COX-2 inhibitors, such as celecoxib, are preferred for patients with a moderate GI risk and with a CV history, owing to their reduced propensity of causing GI complications compared with non-selective NSAIDs [34]. Cardiovascular risk is an equally critical factor in NSAID-prescribing decisions. Patients with established CV disease or high CV risk should avoid non-selective NSAIDs due to their association with increased thrombotic events. Our findings highlight that CV risk was not consistently considered in prescribing practices, underscoring the need for stricter adherence to guideline recommendations that prioritize safer alternatives, such as naproxen or selective COX-2 inhibitors, depending on individual risk profiles. Taken together, the findings highlight that although certain recommendations are being followed, others, in particular those involved in assessing CV risk, are not yet fully integrated into the routine real clinical practice.

Multiple logistic regression analysis reported partial compliance with recommendations concerning the use of non-selective NSAIDs among subjects at high risk for GI and CV issues. The findings indicate that individuals aged 50–64 are prescribed non-selective NSAIDs, which was statistically significant. This raises concerns about deviating from evidence-based practice and therapeutic guidelines and highlights the importance of careful prescribing and adherence to standard prescribing practice, considering the elevated risks of GI and CV adverse effects associated with NSAIDs in older populations [23,26,35].

Comparable trends were noted in those with a history of CV disorders, wherein NSAIDs combined with a gastroprotective agent were more frequently prescribed to individuals with CV diseases. The continued widespread prescribing of NSAIDs in this population remains a recognized concern, given the potential to worsen CV effects. The findings of this study revealed deficiencies in prescriber education concerning the appropriate dangers linked to the prescription of NSAIDs within this vulnerable population. Additionally, the study observed minimal prescription of non-selective NSAIDs, whether used alone or in conjunction with a gastroprotective agent, among moderate-to-high-risk patients. This trend reflects adherence to appropriate prescribing practices as outlined in established NSAID consensus guidelines, including the SER–SEC–AEG multidisciplinary recommendations for the safe prescription of NSAIDs [36]. Similarly, male patients exhibited a notably higher probability of being prescribed preferential COX-2 inhibitors, raising potential concerns regarding GI and CV risks. This signifies gaps in guideline-concordant practice and reinforces the need for prescriber-targeted education [26].

NSAIDs play an essential role in the successful management of pain and inflammation in modern clinical practice. Nonetheless, advocating for the most effective way to evaluate the ‘risks and benefits’ and utilizing this ‘double-edged sword’ accurately for patients continues to be a crucial focus of ongoing research. While NSAIDs hold high therapeutic value, irrational use may also lead to serious drug-related problems, hospital admissions, and healthcare expenditures [37]. Consequently, adopting these treatments in patients with GI risk and a history of CV issues requires frequent monitoring and thorough evaluation of potential side effects. Research indicates that a cautious approach, concordance with the therapeutic guidelines, and utilizing digital health technology tools are effective methods to ameliorate the risk for potential complications [38,39]. Furthermore, providing patients with information regarding the potential risks and benefits associated with NSAID use facilitates informed decision-making, enhances adherence to treatment plans, and enables the recognition of early warning signs of side effects, thereby empowering patients to take a prominent role in self-management [40]. Over-the-counter (OTC) NSAID use was not captured in this study, which may lead to the underestimation of total NSAID exposure and misclassification of GI and CV risks. This represents a potential confounding factor and an area for future research.

The current study has several limitations. As a cross-sectional study, it provides a snapshot of NSAID use and associated GI and CV risks at a single time point, limiting the ability to infer causality or assess long-term outcomes. The use of a consecutive sampling method within a single secondary care hospital may introduce selection bias, as the patient population may not be representative of NSAID users in other healthcare settings or regions. Reliance on electronic health records (EHRs) may also result in missing or incomplete data (e.g., undocumented GI symptoms or CV history), potentially leading to the misclassification of risk categories. Important confounding factors—such as diet, lifestyle, and over-the-counter NSAID use—were not captured and may influence risk assessments. Furthermore, the absence of longitudinal follow-up limits our ability to evaluate how prolonged NSAID exposure affects GI or CV outcomes, particularly among high-risk patients. In addition, although information on prescribed NSAID dose strength was available, the duration of NSAID therapy was not considered in the analysis, as this was not a primary objective of the cross-sectional study, and short prescription durations (e.g., 7–15 days) do not adequately capture long-term gastrointestinal or cardiovascular outcomes. Future multicenter studies with more diverse populations and longitudinal designs are recommended to validate and expand upon these findings.

## 4. Materials and Methods

### 4.1. Study Design and Setting

A prospective, cross-sectional, non-interventional study was conducted in the outpatient (OP) pharmacy unit over six months (January–June 2023) at a secondary care hospital in the northern region of the United Arab Emirates (UAE).

### 4.2. Sample Size and Sampling Technique

The required sample size was calculated by using the Epi Info™ software from the CDC (version 7.2). With a projected total sample size of 1000 patients, the calculation employed a 5% margin of error, a 95% confidence level, and an expected response rate of 50%. A consecutive sampling method was used, where all patients who visited the outpatient pharmacy during the study period and received an NSAID prescription were included.

### 4.3. Ethical Considerations

The principal investigator obtained study approval from the Institutional Ethics Committee (RAKMHSU-REC-031-2022/23-PG-P) and the Regional Research and Ethics Committee [MOHAP/REC/2022/36-2022-PG-P]. The study complied with the ethical guidelines set forth in the Declaration of Helsinki.

### 4.4. Study Criteria

All patients aged 18 years and above, regardless of gender, who received prescriptions containing at least one systemic NSAID, had complete patient data available in the electronic health records (EHRs) of the study location, and granted informed consent, were enrolled in the study. Since the study focused on prescribing patterns rather than clinical outcomes, no minimum duration of NSAID exposure was required.

The study excluded patients receiving care in the emergency room, critical care unit, or inpatient ward; those who received only primary creams, ointments, and compounded products; and those with case records that contained inadequate or missing information.

### 4.5. Study Procedure

All patient prescription records that met the inclusion criteria were included in the study. From the electronic health records (EHRs), the following parameters were extracted: name, age, gender, diagnosis, comorbidities, medication history, and length of hospital stay. A structured data collection form from the outpatient pharmacy department was used to document all medications prescribed to each patient.

GI and CV risk stratifications

Following global prescribing guidelines, eligible patients were categorized based on their cardiovascular (CV) history and gastrointestinal (GI) risk factors [31,35]. CV risk assessment included documented diagnoses such as peripheral arterial disease, stroke, uncontrolled hypertension, acute coronary syndrome, and congestive cardiac failure [41]. Blood pressure readings were reviewed, and values ≥140/90 mmHg during the clinical visit were classified as uncontrolled hypertension [16].

GI risk stratification followed the recommendations of the International NSAIDs Consensus Group [42]. Risk factors included long-term NSAID use; age ≥65 years; comorbid hepatic, renal, metabolic, hypertensive, or CV conditions; concomitant use of aspirin, steroids, anticoagulants, or SSRIs; history of dyspepsia, ulcers, or GI bleeding; and lifestyle factors, such as smoking and chronic alcohol intake [23,43]. The term ‘’need for long-term NSAID use” referred to patients with chronic or recurrent painful conditions—such as arthritis, spondylosis, or chronic musculoskeletal disorders—who required NSAID therapy beyond short-term or episodic use, as described in the GI risk stratification criteria. Helicobacter pylori status was not assessed due to limited data availability.

Patients were categorized into three GI risk groups:

- Minimal risk—no identified risk factors;
- Moderate risk—up to two risk factors;
- High risk—more than two risk factors.

This stratification aligned with previous studies. Based on the International NSAIDs Consensus Group’s guidance, the appropriateness of NSAID prescriptions was assessed. In summary, patients with moderate-to-high GI risk and CV comorbidities should avoid most NSAIDs and may require selective COX-2 inhibitors (e.g., celecoxib) combined with gastroprotective therapy such as PPIs or H2 blockers. Patients with lower GI risk and minimal CV risk may receive non-selective NSAIDs as clinically appropriate.

### 4.6. Data Analysis

IBM SPSS Statistics version 28 was utilized to analyze the data. The demographic information of patients, along with their comorbidities and medication regimens, underwent a thorough descriptive analysis. For continuous variables, the mean and standard deviation are shown. For categorical variables, the frequencies and percentages are shown. We used the chi-square or Fisher’s exact tests to examine the relationship between the risk of GI problems and CV events in individuals using different types of medications. Logistic regression was used to look at the connection between independent variables (like age, gender, relevant patient comorbidities, and GI risk categories) and dependent variables (like the chance of obtaining an analgesic, non-selective COX inhibitors (regardless of a GI protective agent)), or preferential COX-2 inhibitors (irrespective of a GI protective agent). A *p*-value less than 0.05 was judged as significant.

## 5. Conclusions

The current research provides noteworthy observations into NSAID-prescribing patterns in a cohort of subjects with diverse GI and CV risk factors. The findings highlight that although some recommendations were followed, others, in particular those involved in assessing CV risk, remained incompletely integrated into the routine clinical practice. This indicates likely non-adherence to evidence-based therapeutic guidelines and reflects potential gaps in preventive prescribing practices. To ensure safer and more cost-effective treatment options with minimal side effects, especially in at-risk populations, the study strongly recommends a holistic GI and CV risk assessment, close monitoring, and rational co-prescribing practice. Furthermore, the integration of evidence-based digital health tools can help balance the therapeutic benefits of NSAIDs with patient safety in real-world clinical settings. Regular drug utilization studies are essential to ensure that prescribing practices remain aligned with current best practices in clinical care.

As this study represents a snapshot of NSAID prescribing in a single hospital, the generalizability of the findings is limited; however, it provides valuable insight into actual prescribing behavior within the studied healthcare context.

## Figures and Tables

**Table 1:** Socio-demographic characteristics of the study population.

Variable	*N* = 1005 (%)	95% Confidence Interval
Gender		
• Female	535 (53.23)	50.0–56.6
• Male	470 (46.8)	43.4–50.0
Age (In Years)		
• ≤25	92 (9.15)	7.3–10.9
• 26–50	509 (50.64)	47.6–54.0
• 51–75	364 (36.21)	33.3–39.4
• >75	40 (3.98)	2.9–5.3
Nationality		
• UAE Nationals	718 (71.40)	68.7–74.0
• Expatriates	287 (28.55)	26.0–31.3
Number of comorbidities		
• 0	474 (47.16)	44.2–50.3
• 1	241 (23.98)	21.3–26.7
• 2	175 (17.41)	15.0–19.8
• 3	76 (7.56)	6.0–9.3
• 4	32 (3.18)	2.1–4.3
• 5	06 (0.59)	0.2–1.1
• 6	01 (0.09)	0.0–0.3
Comorbidities		
Diabetes mellitus	201 (20.0)	77.5–82.5
CV diseases	235 (23.4)	20.7–26.1
Dyslipidemia	143 (14.2)	11.9–16.5
Thyroid disorders	55 (5.5)	4.1–7.0
GI diseases	57 (5.7)	4.3–7.2
Kidney diseases	12 (1.2)	0.6–1.9
Anemia	61 (6.1)	4.7–7.7
Respiratory	58 (5.8)	4.4–7.3
Musculoskeletal	45 (4.5)	3.3–5.8
Neurological	24 (2.4)	1.4–3.8
Psychiatric	37 (3.7)	2.6–4.9
Dermatological	19 (1.9)	1.1–2.7
Others	970 (96.5)	95.4–97.7
Total No. of Prescription Drugs		
1–5	736 (73.6)	70.3–75.8
6–10	216 (21.6)	19.2–24.2
11–15	43 (4.3)	3.1–5.5
>16	10 (1.0)	0.5–1.6

Confidence intervals (CI) indicate the estimated range of values within which the true population parameter is likely to fall with 95% confidence.

**Table 2:** Clinical indication category for the use of NSAIDs.

Sl No.	Clinical Indication	Frequency (*n* = 1210)	%
1	Musculoskeletal and mechanical pain	750	61.98
2	Inflammatory joint disorders	143	11.81
3	Dental and ENT-related pain	175	14.46
4	Acute traumatic conditions	139	11.48
5	Chronic disease-related pain	43	3.55
6	Neuropathic pain	11	0.9

**Table 3:** Patterns of NSAID prescriptions according to therapeutic class, prescribed dose, and frequency of administration.

Classification	ATC Code	Prescribed Dose (In mg)	Frequency of Administration	Number of NSAIDs Prescriptions (*n* = 2051)	%
**Analgesic/Antipyretic with Poor Anti-Inflammatory Action**					
Paracetamol (*n* = 474)	N02BE01	500 mg	One tablet sixth hourly	77	3.75
			One tablet twice daily	115	5.60
			One tablet thrice daily	26	1.26
			One tablet twice daily (with Orphenadrine citrate)	256	12.48
**Non-selective COX inhibitors**					
Aspirin (*n* = 31)	B01AC06	81 mg	One tablet daily	08	0.39
		100 mg	One tablet daily	23	1.12
Ibuprofen (*n*= 142)	M01AE01	400 mg	One tablet daily	04	0.19
			One tablet twice daily	61	2.97
			One tablet three times a day	68	3.31
		200 mg	One tablet daily	02	0.09
			One tablet twice daily	07	0.34
Indomethacin (*n* = 31)	M02AA23	8 mg/mL spray	One application twice daily	31	1.51
Ketoprofen (*n* = 399)	M02AA10	25 mg/g (2.5% gel)	One application twice daily	241	11.75
			One application thrice daily	158	7.70
Piroxicam (*n* = 112)	M01AC01	20 mg	Once daily	106	5.16
			Twice daily	06	0.29
**Preferential COX-2 Inhibitors**					
Diclofenac (*n* = 226)	M01AB05	50 mg	Once daily	17	0.82
			Twice daily	34	1.65
			Thrice daily	01	0.04
		Topical gel 1%	Once a day	05	0.24
			Twice daily	143	6.97
			Thrice daily	03	0.14
			Four times a day	23	1.12
Meloxicam (*n* = 149)	M01AC06	7.5 mg	Once daily	104	5.07
		15 mg	Twice daily	45	2.19
**Selective COX-2 inhibitors**					
Celecoxib (*n* = 487)	M01AH01	200 mg	Once daily	273	13.31
			Twice daily	214	10.43

**Table 4:** Gastrointestinal (GI) risk factors among the study population and their association with NSAID use.

GI Risk Factors	Frequency * (*n* = 1005)	Percentage	*p* Value
Need for long-term NSAID use	490	48.75	0.018 ^†^
Age (>65 years)	141	14.02	<0.0001 ^†^
Comorbidities			
*CV*	224	22.28	0.005
*Renal*	12	1.19	0.756 ^†^
*GI*	54	5.37	0.137 ^†^
*Rheumatological*	42	4.17	0.079 ^†^
Long-term smoking habit	43	4.27	0.733 ^†^
Chronic intake of alcohol	None	--	--
History of steroid use	08	0.79	0.830 ^†^
Aspirin use	31	3.08	0.483 ^†^
Anticoagulant use	04	0.39	<0.0001 ^†^
Selective serotonin reuptake inhibitor (SSRI) use	16	1.59	0.328 ^†^

* Chi-square test was used for analysis, and *p* ≤ 0.05 was considered significant; *n* = Number of prescriptions, NSAID—Nonsteroidal anti-inflammatory drug; ^†^ Fisher’s exact test.

**Table 5:** Assessment of drug therapy according to the level of the patient’s GI risks.

Types of Drugs	Low GI Risk	Moderate Risk	High Risk	*p* Value
	*N* = 763 (%)	*N* = 204	*N* = 38	
Paracetamol	335 (43.90)	103 (50.49)	18 (47.36)	0.236
NsNSAIDs	219 (28.70)	47 (23.03)	24 (63.15)	<0.0001
NsSAIDs with PPI	49 (6.42)	15 (7.35)	18 (47.36)	<0.0001
Preferential COX-2 Inhibitors	236 (30.93)	78 (38.23)	17 (44.73)	0.041
Preferential COX-2 Inhibitors with PPI	105 (13.76)	36 (17.64)	10 (26.31)	0.052
Selective COX-2	382 (50.06)	103 (50.49)	14 (36.84)	0.275
Selective COX-2 with PPI	184 (24.11)	50 (24.50)	10 (26.31)	0.946

NsNSAIDs—non-selective NSAIDs; PPI—proton-pump inhibitors; COX-2—cyclooxygenase-2 inhibitors; *p* < 0.05—statistically significant. *p* < 0.0001 indicates a statistically highly significant difference in NSAID-prescribing patterns across the GI risk categories.

**Table 6:** Univariate analysis of the prescribing of NSAIDs and GI and CV risks of patients.

Type of Drug	Low GI Risk		Moderate GI Risk		High GI Risk		*p* Value
	No CV Hx (*n* = 651) (%)	CV Hx (*n* = 112) (%)	No CV Hx (*n* = 104) (%)	CV Hx (*n* = 100) (%)	No CV Hx (*n* = 11) (%)	CV Hx (*n* = 27) (%)	
Paracetamol	279 (42.85)	56 (50.0)	53 (50.96)	50 (50.0)	05 (45.45)	13 (48.14)	0.433
NsNSAIDs	188 (28.87)	31 (27.67)	26 (25.0)	21 (21.0)	8 (72.72)	16 (59.25)	<0.0001
NsSAIDs with PPI	34 (5.22)	15 (13.39)	5 (4.80)	10 (10.0)	6 (54.54)	12 (44.44)	<0.0001
Preferential COX-2 Inhibitors	200 (30.72)	36 (32.14)	41 (39.42)	37 (37.0)	5 (45.45)	12 (44.44)	0.252
Preferential COX-2 Inhibitors with PPI	89 (13.67)	16 (14.28)	20 (19.23)	16 (16.0)	4 (36.36)	6 (22.22)	0.180
Selective COX-2	324 (49.76)	58 (51.78)	48 (46.15)	55 (55.0)	02 (18.18)	12 (44.44)	0.260
Selective COX-2 with PPI	157 (24.11)	27 (24.10)	23 (22.11)	27 (27.0)	01 (9.09)	09 (33.33)	0.663

NsNSAIDs—non-selective NSAIDs; PPI—proton pump Inhibitors; COX-2—cyclooxygenase-2 inhibitors; *p* < 0.05—statistically significant.

**Table 7:** Multivariate logistic regression analysis of the association between patients’ characteristics and the prescription of nonselective NSAIDs, preferential cyclooxygenase, and selective cyclooxygenase-2 inhibitors.

Variables	Paracetamol	NsNSAIDs	NsNSAIDs with PPI	Preferential COX-2 Inhibitors	Preferential COX-2 Inhibitors with PPI	Selective COX 2	Selective COX2 with PPI
Age < 50	Reference	Reference	Reference	Reference	Reference	Reference	Reference
50–64	0.683 (0.284–1.639)	3.422 * (1.261–9.289)	2.715 (0.635–11.612)	1.060 (0.441–2.549)	0.955 (0.337–2.703)	1.010 (0.422–2.414)	1.948 (0.678–5.594)
65–74	1.107 (0.467–2.622)	2.368 (0.881–6.363)	3.294 (0.800–13.568)	0.876 (0.368–2.087)	1.086 (0.389–3.028)	1.428 (0.605–3.374)	2.433 (0.860–6.883)
≥75	1.732 (0.838–3.580)	1.037 (0.434–2.479)	1.076 (0.328–3.526)	1.016 (0.492–2.101)	0.633 (0.265–1.508)	1.970 (0.947–4.098)	1.841 (0.736–4.603)
Gender (male)	0.789 (0.612–1.016)	0.874 (0.660–1.157)	0.791 (0.485–1.288)	1.431 * (1.097–1.868)	1.258 (0.887–1.785)	0.718 * (0.558–0.923)	0.724 * (0.540–0.972)
CVS	0.937 (0.661–1.329)	1.104 (0.743–1.640)	2.363 * (1.301–4.289)	1.103 (0.764–1.591)	0.935 (0.580–1.508)	1.092 (0.771–1.547)	1.108 (0.747–1.643)
Neurology	0.388 * (0.149–1.000)	0.782 (0.309–1.979)	0.000 (0.000–0.012)	1.052 (0.465–2.380)	0.954 (0.330–2.760)	1.229 (0.560–2.697)	1.557 (0.668–3.629)
GIT	1.284 (0.691–2.383)	0.702 (0.352–1.399)	0.556 (0.196–1.574)	1.121 (0.594–2.117)	0.689 (0.297–1.598)	1.024 (0.551–1.902)	1.015 (0.508–2.029)
Rheumatology	1.534 (0.819–2.876)	0.755 (0.356–1.598)	0.166 (0.021–1.313)	0.621 (0.305–1.265)	0.492 (0.171–1.420)	1.366 (0.728–2.562)	1.405 (0.727–2.715)
CKD	3.146 (0.834–11.875)	2.005 (0.591–6.794)	1.603 (0.274–9.375)	0.333 (0.071–1.562)	0.476 (0.060–3.782)	0.682 (0.211–2.207)	1.026 (0.270–2.715)
Low GI	Reference	Reference	Reference	Reference	Reference	Reference	Reference
Moderate GI	1.720 (0.657–4.498)	0.078 ** (0.027–0.219)	0.035 ** (0.009–0.130)	0.564 (0.215–1.476)	0.271 * (0.087–0.844)	2.847 * (1.077–7.524)	0.796 (0.273–2.319)
High GI	1.334 (0.633–2.809)	0.122 ** (0.054–0.274)	0.061 ** (0.023–0.165)	0.792 (0.377–1.664)	0.504 (0.211–1.202)	2.019 (0.942–4.328)	0.872 (0.376–2.023)

COX-2—cyclo-oxygenase-2; GI—GI; PPI—proton-pump inhibitor; NsNSAIDs—nonselective nonsteroidal anti-inflammatory drugs; CVS—CV system; CKD—chronic kidney disease; ** *p* < 0.0001; * *p* < 0.05.

## Data Availability

The data supporting the findings of this study are available from the corresponding author upon reasonable request.

## Abbreviations

The following abbreviations are used in this manuscript:

NSAIDs	Non-steroidal anti-inflammatory drugs
pDDIs	Potential drug–drug interactions
COX-2	Cyclooxygenase-2
PPIs	Proton-pump inhibitors
UAE	United Arab Emirates
CV	Cardiovascular
GI	Gastrointestinal
